# Effects of body positions and anaesthesia on the distribution of ventilation in alpacas (*Vicugna pacos*)

**DOI:** 10.3389/fvets.2026.1771457

**Published:** 2026-04-13

**Authors:** Andrea Basler, Sonja Franz, Ulrike Auer, J. Peter Schramel, Remco Folkertsma, Andreas D. Waldmann, Martina Mosing

**Affiliations:** 1 Anaesthesiology and Intensive-Care, Clinical Centre for Small Animal Health and Research, Clinical Department for Small Animals and Horses, University of Veterinary Medicine Vienna, Vienna, Austria; 2 Clinical Centre for Ruminants, Camelids and Herd Health Management, Clinical Department for Farm Animals and Food System Transformation, University of Veterinary Medicine Vienna, Vienna, Austria; 3 Platform for Bioinformatics and Biostatistics, Department of Biological Sciences and Pathobiology, University of Veterinary Medicine Vienna, Vienna, Austria; 4 Department of Anaesthesiology and Intensive Care Medicine, Rostock University Medical Centre, Rostock, Germany

**Keywords:** alpaca, electrical impedance tomography, ventilation distribution, anaesthesia, body position

## Abstract

This study monitored and detected changes in the distribution of ventilation and other electrical impedance tomography (EIT) related ventilation variables across different body positions throughout all peri- and intra-anaesthetic stages in alpacas, while also evaluating intra-individual differences between two anaesthetic procedures. Six healthy adult male alpacas underwent two anaesthetic sessions to assess ventilation using EIT across various body positions, including awake standing (Stand_pre_), sternal sedated (sternal_sed_), during controlled mechanical ventilation in sternal with different head positions (Sternal_CMVhigh_, Sternal_CMVlow_), sternal spontaneously breathing with tube (Sternal_spontETT_), sternal without tube (Sternal_spont_), and post-anaesthesia standing (Stand_post_). Sedation and anaesthesia were performed with ketamine, xylazine, and butorphanol, and maintained with isoflurane. EIT data were collected over 2 to 5 min, and six to ten consecutive artefact-free breaths per phase were analysed for EIT-related ventilation variables. Statistical analysis was performed using linear mixed-effects models (significance at *p* < 0.05). The centre of ventilation along the right-left axis did not differ across phases [*F*_(6, 66.08)_ = 1.33, *p* = 0.257], whereas ventral-dorsal values varied significantly [*F*_(6, 67.19)_ = 3.92, *p* = 0.002]. Regional ventilation remained stable throughout all phases [*F*_(6, 66.06)_ = 1.30, *p* = 0.269]. Tidal rate showed a significant overall effect of phase [*F*_(6, 72.00)_ = 20.43, *p* < 0.001], with the highest tidal rate observed during Stand_pre_ compared with other phases. Tidal impedance variation showed a significant overall difference [*F*_(6, 67.08)_ = 3.21, *p* = 0.007], despite no significant *post-hoc* comparisons. Minute impedance variation (MIV) differed across phases [*F*_(6, 67.05)_ = 23.58, *p* < 0.001], and inspiratory time had a significant overall impact on the phases [*F*_(6, 67.30)_ = 11.37, *p* < 0.001]. End-expiratory lung impedance remained stable over all phases [*F*_(6, 67.05)_ = 1.89, *p* = 0.094]. Alpacas appeared to maintain a relatively stable ventilation distribution with small changes across body positions and anaesthetic phases. This may reflect the influence of their distinctive lung, diaphragm, and gastrointestinal anatomy. Changes from standing to sternal positions minimally affect ventilation parameters, and sedation with xylazine, ketamine, and butorphanol modify some of these variables without compromising overall respiratory performance.

## 1 Introduction

It is known that the distribution of ventilation and respiratory pattern can be markedly influenced by changes in body position across multiple species (1–8). Similarly, the administration of sedative or anaesthetic agents has been shown to exert significant effects on tidal volume, respiratory rate, and functional residual capacity (FRC), among other parameters (9–11). The transition from standing to sternal recumbency, commonly achieved through the use of such drugs, leads to a shift of abdominal organs pushing on the diaphragm that may further compromise ventilatory efficiency. This phenomenon can be particularly relevant in ruminants, in which diaphragmatic compression may occur secondary to ruminal distension (4). Besides positioning, the transition from spontaneous to controlled mechanical ventilation (CMV) during maintenance of anaesthesia changes the distribution of ventilation by suppressing physiologic motion of the diaphragm (6, 12, 13).

Although alpacas are not true ruminants, the bulk of their gastrointestinal anatomy within the abdomen is similar to that of ruminants. In contrast, their gross pulmonary anatomy is more comparable to that of horses (11). Despite this, information regarding respiratory function in camelids remains very limited compared with that available for other domestic species (11).

Thoracic electrical impedance tomography (EIT) has been performed in awake, sedated, or anaesthetised human and veterinary patients, providing flexibility across different clinical scenarios (14). It is a non-invasive imaging modality that enables real-time assessment of regional impedance changes within the lungs. The technique employs an electrode belt positioned around the thorax, through which a low-amplitude alternating current is applied. While the current is applied through one pair of electrodes, voltage differences are measured across the remaining electrodes. Variations in impedance are then used to assess ventilation parameters and detect dynamic changes as they occur (14–16). EIT has been utilised to characterise ventilation distribution during different interventions in a variety of species, as well as to detect pathological processes without the need for additional imaging modalities (17–23).

The aim of this study was to evaluate thoracic electrical impedance tomography (EIT) changes occurring during the peri-anaesthetic period in alpacas, systematically divided into distinct and well-defined phases: (1) in the awake standing position, (2) during sedation in sternal recumbency, sternal under general anaesthesia with mechanical ventilation with, (3) head high and (4) head low positions, (5) sternal while breathing spontaneously with an endotracheal tube, (6) sternal without an endotracheal tube, and finally (7) in the standing position after complete recovery. In addition, this crossover study aimed to assess intra-individual differences between two identical anaesthetic procedures separated by a 7-week washout period. We hypothesised that the transition from awake standing to sedated sternal recumbency and subsequently to CMV will produce a redistribution of ventilation from dependent toward non-dependent lung regions. In addition, compared with the awake standing baseline, sedation in sternal recumbency is expected to decrease respiratory rate and the EIT variables, which are surrogates for minute volume and FRC.

## 2 Materials and methods

The present study was approved by the Institutional Ethics Committee of Vetmeduni Vienna, Austria, and received governmental authorization (GZ 2023-0.896.923). The manuscript adheres to the ARRIVE 2.0 guidelines (24). This investigation was conducted concurrently with data collection on regional cerebral oxygen saturation using near-infrared spectroscopy (NIRS), as part of a broader research project.

### 2.1 Animals

This experimental crossover study included six healthy, adult, non-castrated male alpacas with a median age of 2.9 (2.6–8.8) years, and a median body weight of 70 (49.6–88.4) kg. Animals younger than 2 years or older than 10 years were excluded.

The alpacas were acclimatised to their housing for 48 h prior to the experiment. Food was withheld for 8 to 12 h before the procedure, but all animals had free access to water until premedication. All animals underwent a complete clinical examination, including respiratory assessment by thoracic auscultation, evaluation of respiratory rate and pattern, inspection for abnormal respiratory signs, and assessment of mucous membrane colour and capillary refill time. Haematology and serum biochemistry tests were also performed, after which all animals were classified as ASA physical status I.

Each alpaca underwent two anaesthetic events to test the repeatability of EIT data with a 7-week washout period between sessions. During the first anaesthetic event, all animals had their normal long fleece, whereas during the second event they had undergone their annual shearing.

### 2.2 Anaesthesia

Following EU legislation on food-producing animals, all animals were premedicated via intramuscular injection (IM) with a combination of xylazine (0.3 mg/kg; Sedaxylan^®^ 20 mg/ml, Dechra Veterinary Products GmbH, Germany), ketamine (3 mg/kg; Ketamidor^®^ 100 mg/ml, Richter Pharma AG, Wels, Austria), and butorphanol (0.2 mg/kg; Butomidor^®^ 10 mg/ml, Richter Pharma AG, Wels, Austria), administered in a single syringe. If sternal recumbency was not achieved within 5 min, an additional IM dose of ketamine (1 mg/kg) and xylazine (0.2 mg/kg) was administered.

Once in sternal recumbency, an intravenous 20-gauge catheter (Vasofix^®^ Safety, B. Braun Melsungen AG, Melsungen, Germany) was placed in the cephalic vein, and one auricular artery was catheterised with a 22-gauge catheter (Vasofix^®^ Safety, B. Braun Melsungen AG, Melsungen, Germany) for invasive blood pressure monitoring.

Anaesthesia was induced by repeated intravenous administration of ketamine (0.5 mg/kg) until loss of consciousness, defined as absence of the palpebral reflex, loss of muscle tone, and swallowing reflex at the time of intubation. A total dose of 2.5–3 mg/kg was required in all animals to permit tracheal intubation with a cuffed endotracheal tube (MarMed, ∅ 10.0 mm / ∅ 11.0 mm; MarMed GmbH, Germany). The tube was attached to a circle system, and controlled mechanical ventilation (CMV) was applied using an Ohmeda 7,800 ventilator (GE Healthcare, Finland). Anaesthesia was maintained with isoflurane (Vetflurane^®^1,000 mg/g, Virbac S.A, France) in 100% oxygen. Isoflurane concentration was maintained depending on the need to ensure an adequate depth of anaesthesia, defined as unconsciousness and the absence of a response to external stimuli. Standard anaesthetic cardiopulmonary variables were continuously monitored and manually recorded every 5 min during the anaesthetic phase, using a multiparametric monitor (Carescape B650, GE Healthcare, Finland). Fluids (Sterofundin^®^ ISO Infusionslösung, B. Braun Melsungen AG, Germany) were administered throughout the procedure with an infusion pump (at a rate of 10 ml/kg/hour IV).

### 2.3 Mechanical ventilation

Throughout the duration of anaesthetic gas administration, all alpacas were maintained on volume-controlled mechanical ventilation. Ventilation parameters were adjusted to maintain an end-tidal CO_2_ (EtCO_2_) of 35–45 mmHg.

Initial settings included a tidal volume of 10 mL/kg, a respiratory rate of 10 breaths per minute, and a positive end-expiratory pressure (PEEP) of 5 cmH_2_O. If the target EtCO_2_ was not achieved, adjustments were made first by increasing the respiratory rate by one breath per minute up to a maximum of 18 breaths per minute and, if necessary, by increasing the tidal volume stepwise by 2 ml/kg. The PEEP was kept constant throughout the study. The EtCO_2_ measurements were confirmed by arterial blood gas analysis every 10 min throughout anaesthesia.

Patient–ventilator asynchrony was assessed qualitatively during the mechanical ventilation phases by evaluating ventilator waveforms (flow, pressure, and volume curves), thoracoabdominal movements, and EIT signals. Asynchrony was defined as a lack of coordination between ventilator-delivered breaths and the animal's breathing efforts, identified by inconsistencies between EIT-derived ventilation patterns and ventilator cycles. Due to the exploration nature of this assessment and the small sample size, no formal quantitative scoring system was applied.

### 2.4 Electric impedance tomography (EIT)

#### 2.4.1 Computed tomography (CT) and finite element (FE) model

Prior to commencing the study, the EIT belt position was pre-defined based on existing thoracic CT scans of alpacas not involved in this research as follows: To evaluate pulmonary tissue, the region with minimal interference from the cardiac silhouette and abdominal organs was selected. After reviewing the images, the landmarks in standing alpacas were defined at the mid-level of the fifth intercostal space, measured vertically to the floor. To confirm the correct belt positioning, a sham belt with radiodense markers was placed on two alpacas, which required CT scans as part of their diagnostic evaluation. Computed tomography images of the thorax were acquired in sternal recumbency using a Siemens Healthineers Somatom X.cite 128 CT scanner, with a slice thickness of 0.8 mm and a slice interval of 0.6 mm (0.8 × 0.6 mm). The CT slides obtained with the sham belt in position were used to create the alpaca thorax finite element model.

**Figure 1:**
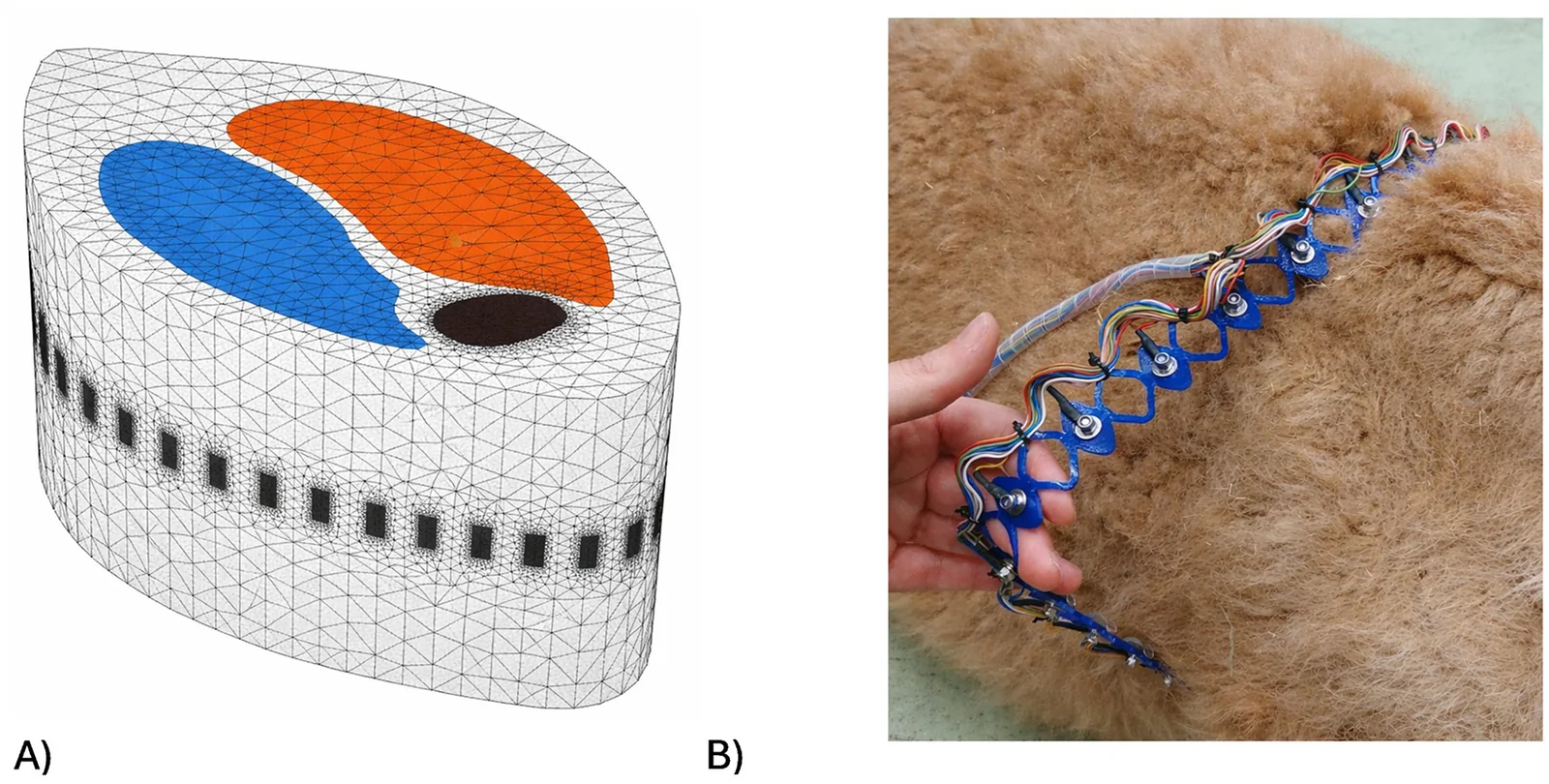
**(A)** Finite element model of the alpaca thorax. The regions of interest are highlighted: blue = right lung, red = left lung, and black = heart. Black squares indicate the positions of the electrodes. **(B)** Custom-made EIT belt positioned at the level of the fifth intercostal space (mid-thoracic level) in an alpaca.

Using a dedicated software (ITK-SNAP) (25) the heart, lungs, and thoracic cavity were manually segmented from the corresponding DICOM. The segmented contours were then used to build an animal-specific finite element (FE) model (Figure 1).

#### 2.4.2 EIT data collection

A custom-made EIT belt was used, matching the required chest circumference of alpacas (80–110 cm). The belt consisted of 32 electrodes made of M4 x 15 mm stainless steel washers. Two sides were bent 90° to form two ridges that extend through the fleece, ensuring good skin contact. The electrodes were mounted equidistantly with M3 stainless steel flat-head screws on a custom-made elastic structure, 3D-printed from the flexible filament TPU-95 (thermoplastic polyurethane) (Figure 1). All electrodes were connected via a custom-made connector box to the EIT Lumon connector and monitor.

The fleece was parted along the previously identified anatomical landmarks to expose the skin as much as possible. An electrically low-conductivity ultrasound gel was generously applied to both the electrodes and the thoracic skin to ensure optimal conductivity. The belt was then placed around the chest and wrapped with an elastic bandage to improve contact pressure, prevent gel from drying, and to minimize belt movement.

Proper electrode contact was verified using the built-in function of the EIT software, which visually indicated successful or failed connections. For data acquisition, the EIT belt was connected to the Lumon device (SenTec, Switzerland).

### 2.5 Study design

This study was a prospective, crossover experimental design randomized for one position (head high/low), with repeated measurements in six healthy adult male alpacas. EIT was used to assess lung ventilation across multiple anaesthetic phases and body positions, combining observational monitoring with controlled interventions including anaesthesia drug administration, mechanical ventilation, and postural changes.

#### 2.5.1 Standing pre-anaesthesia (Stand_pre_)

**Figure 2:**
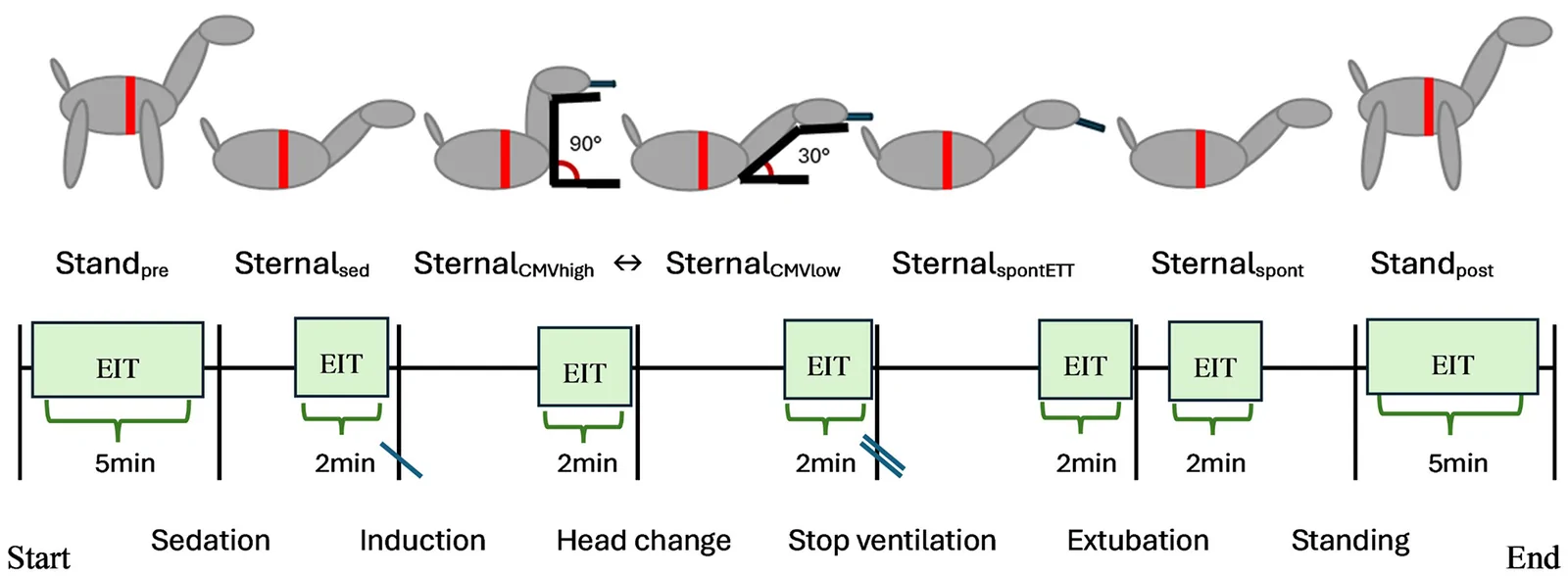
Timeline. Time points corresponding to different body positions and measurement phases (awake, sedation, and anaesthesia) in six alpacas, from which six to ten consecutive breaths were collected for EIT analysis. Stand_pre_, Standing pre-sedation; Sternal_sed_, Sternal recumbency after sedation; Sternal_CMVhigh_, Sternal recumbency controlled mechanical ventilation head high; Sternal_CMVlow_, Sternal recumbency controlled mechanical ventilation head low; Sternal_spontEIT_, Sternal recumbency spontaneous breathing with endotracheal tube; Sternal_spont_, Sternal recumbency spontaneous breathing without endotracheal tube; Stand_post_, Standing post anaesthesia; \, start anaesthesia; \\, end anaesthesia; ↔, randomized.

After confirming sufficient contact of all electrodes, a five-minute period of EIT data collection was started in a standing position (Figure 2). The animal was held with the neck and head straight, with a leash attached to a head holster in a quiet environment. Then the EIT belt was disconnected again from the EIT device without removing the belt, and the alpacas were sedated.

#### 2.5.2 Sternal recumbency during sedation (Sternal_sed_)

Once the alpacas were sedated and positioned in sternal recumbency, their head and necks were supported to maintain them in a physiological position. EIT data were recorded for 2 min following 10 min in sternal recumbency (Figure 2).

#### 2.5.3 Anaesthesia phase: sternal recumbency during controlled mechanical ventilation with head high and low (Sternal_CMVhigh_ and Sternal_CMVlow_)

After induction of anaesthesia and ETT placement, CMV was initiated using the settings described above. The neck and head of the alpacas were positioned in a custom-made metallic frame that allowed the head to be held in either a high or low posture. The high-head (CMVhigh) position was defined as a 90° angle between the frame and the ground, whereas the low-head (CMVlow) position corresponded to a 30° angle relative to the ground (Figure 2). During the first anaesthesia, each animal was randomly assigned to start in either the low or high head position, which was then changed after 30 min to the opposite position. During the second anaesthesia, each animal commenced in the position opposite to that used in the first session, and the procedure was repeated. In both anaesthetic events, after 28 min with the initial head position, a 2-min EIT recording was obtained, and the head position was changed at 30 min. The same procedure was then repeated for the opposite head position.

#### 2.5.4 Sternal recumbency during spontaneous breathing with an endotracheal tube (Sternal_spontETT_)

After finalizing the last measurement, isoflurane delivery was discontinued, and the alpacas were allowed to breathe spontaneously. The animal's head and neck were maintained in the metallic frame at a 30° angle until extubation was performed. EIT data were recorded during the 2 min preceding extubation. ETT was removed when alpacas showed clear signs of readiness for extubation, such as swallowing or chewing movements.

#### 2.5.5 Sternal recumbency during spontaneous breathing without an endotracheal tube (Sternal_spont_)

Two minutes after extubation, EIT was recorded for 2 min with the animal in sternal recumbency. The head and neck were supported to maintain them in a physiological position until the animals regained the ability to hold their head unassisted (Figure 2).

#### 2.5.6 Standing post-anaesthesia (Stand_post_)

Once the animals had regained a standing posture and were able to maintain their necks in a physiological straight position, a 5-min EIT data recording period was started (Figure 2).

### 2.6 *Post-hoc* data analysis

The raw EIT data were retrospectively analysed using ibeX software (SenTec, Switzerland). For each alpaca and during each position, EIT data from six to ten consecutive artefact-free breaths were analysed. EIT recordings were obtained under both anaesthesia, resulting in two datasets per animal for each analysed phase. From the selected breaths, the following EIT variables were exported and analysed:

1. **Centre of Ventilation (CoV)**
  CoV represents the focal point of overall ventilation expressed as a % value. For right–left distribution, a CoV_RL_ of 0% indicates that the focal point lies in the outermost region of the right lung, whereas a value of 100% corresponds to the outermost region of the left lung. Similarly to the right–left ventilation, the ventral–dorsal centre of ventilation (CoV_VD_) reflects the focal point of ventilation along this axis. A CoV_VD_ of 0% corresponds to the most ventral region of the lung, whereas 100% corresponds to the most dorsal region (6, 14).
2. Region of interest right and left lung
  The EIT image is divided by a vertical line through the anatomical midline into a right and a left region of interest. Impedance changes in both lung areas are analysed.
3. **Tidal rate**
  The tidal rate corresponds to the respiratory rate, representing the number of breaths taken per minute
4. **Tidal Impedance Variation (TIV** **=** **ΔZ)**
  TIV represents the total change in lung impedance between the beginning and end of inspiration for each breath and can serve as a surrogate for tidal volume. It is calculated by subtracting the impedance (Z) at the end of inspiration from the impedance at the beginning (ΔZ). The term TIV is used when the impedance change is expressed in arbitrary units (AU).
5. **Minute impedance variation (MIV)**
  MIV is a surrogate for minute ventilation, representing the total lung impedance change over 1 min. It is calculated by multiplying the tidal rate with TIV
6. **Inspiratory time (TI)**
  TI measures the duration of the inspiratory phase of a breath, expressed in seconds (s). It is calculated by subtracting the end of inspiration from the start of inspiration.
7. **End-expiratory lung impedance (EELI)**
  EELI reflects the electrical impedance of the lungs at the end of expiration and provides an approximate measure of lung volume at that point in the respiratory cycle and therefore is a surrogate for functional residual capacity (FRC) (14).

### 2.7 Statistics

A formal sample size calculation was not conducted, as this study was a complementary investigation to an ongoing study that included a power analysis. Due to the lack of prior data on EIT-derived parameters in this species, no reliable estimates of variance or effect size were available to support a meaningful a priori calculation. Ethical considerations and adherence to the 3Rs principle further supported the use of the same limited number of animals.

To estimate if EIT variable values differed across phases, a series of linear mixed models (26) was used. In each of these models, the respective variable was included as the response, phase as the fixed effect predictor, and subject as the random effect. After fitting each model, it was confirmed that none of the model assumptions regarding normally distributed and homogeneous residuals were violated by visual inspection of QQ-plots of residuals and residuals vs. fitted plots. The overall significance of phase was tested by means of the Satterthwaite approximation (27) using the function lmer of the package lmerTest (version 3.1-3) (28) and a model fitted with restricted maximum likelihood. *Post-hoc* pairwise comparisons of the effect of phase were applied using the emmeans package (version 1.7.2) (29). Repeatability of measurements across both measurements was estimated by calculating the intra-class correlation coefficient (ICC) (30) using the ICC function in the package irr (version 0.84.1) (31) with the model argument set to “twoway,” type to “agreement,” and unit to “single.” Figures were created using the package ggplot (32). All analyses were conducted in R (version 4.5.1) (33).

## 3 Results

All six alpacas underwent two identical anaesthetic procedures, during which EIT data were collected at the designated time points (see Figure 2). In one alpaca, the last four phases (Sternal_CMVlow_, Sternal_spontETT_, Sternal_spont_, Stand_post_) could not be recorded due to a technical problem with the software of the device. In another alpaca, the final phase (Stand_post_) was not recorded due to the lack of cooperation of the animal. General anaesthesia was uneventful in all animals, with all cardiopulmonary variables, including blood gases, remaining within normal physiological ranges. The belt was applied without complications, achieving a consistent and proper interface between the skin and the device in every case. After removal of the belt, any residual ultrasound gel was carefully removed with water, and no alterations of the fleece or signs of skin redness were observed.

### 3.1 CoV

**Figure 3:**
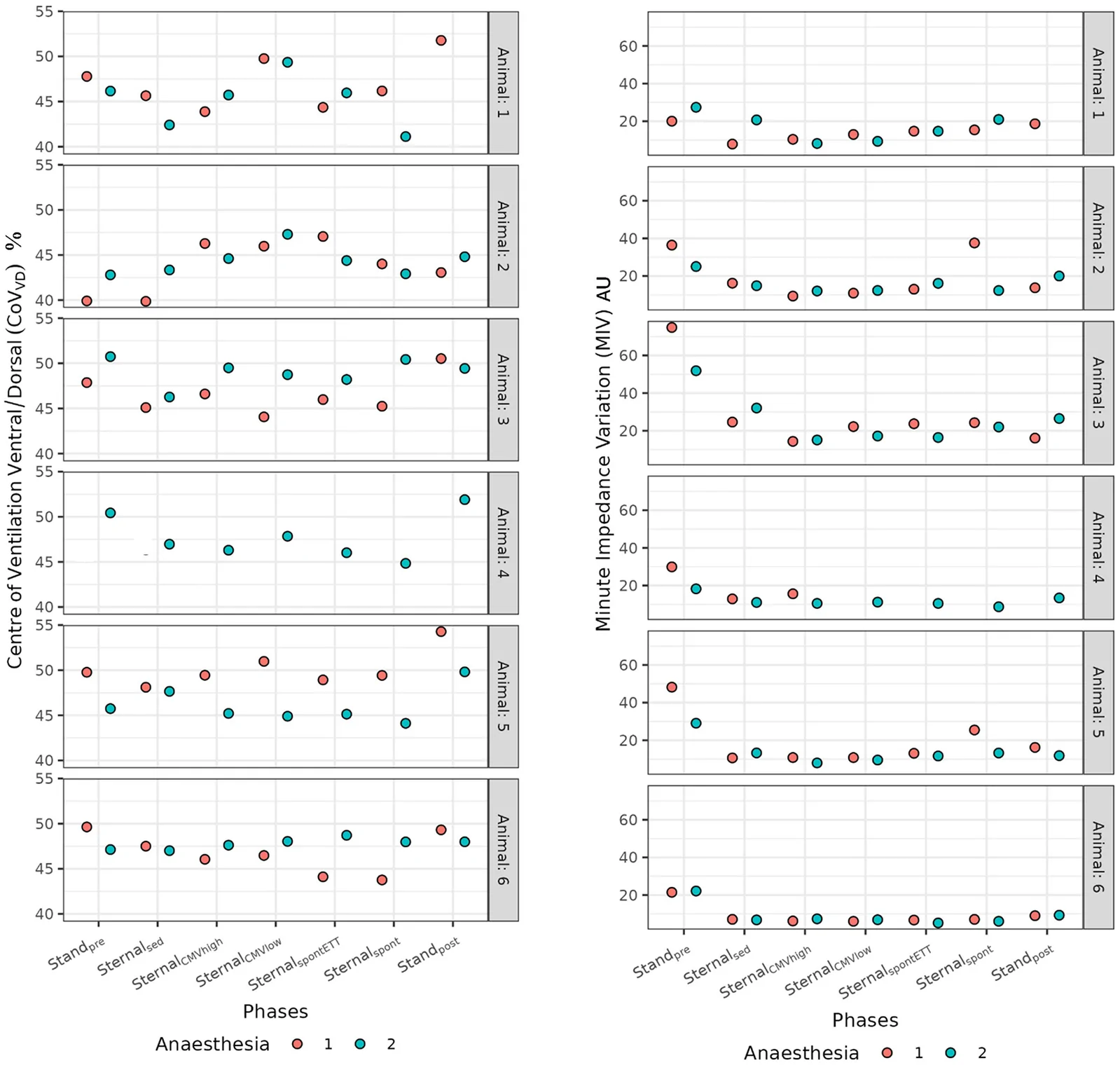
Centre of ventilation (ventral-dorsal) and minute impedance variation displayed individually for six alpacas across all experimental phases, including sedation, controlled mechanical ventilation (head high/low), spontaneous breathing, and post-anaesthesia, during two EIT measurement events. The x-axis represents the experimental phases: Stand_pre_, Standing pre-sedation; Sternal_sed_, Sternal recumbency after sedation; Sternal_CMVhigh_, Sternal recumbency controlled mechanical ventilation head high; Sternal_CMVlow_, Sternal recumbency controlled mechanical ventilation head low; Sternal_spontEIT_, Sternal recumbency spontaneous breathing with endotracheal tube; Sternal_spont_, Sternal recumbency spontaneous breathing without endotracheal tube; Stand_post_, Standing post anaesthesia. The y-axis shows the centre of ventilation ventral/dorsal in percentage (%) and minute impedance variation in arbitrary units (AU) per minute, respectively. Anaesthesia 1, first EIT measurement event (red dot); Anaesthesia 2, second EIT measurement event (green dot); Animal 4 anaesthesia excluded due to technical difficulties.

No significant differences in mean CoV_RL_ were detected between the evaluated body positions [*F*_(6, 66.08)_ = 1.33, *p* = 0.257] (Table 1). The overall mean CoV_RL_ during the study period was 49.2 ± 2.8%. The CoV_VD_ was consistently located in the ventral region across all positions, with a mean of 46.75 ± 2.51%. Analysis showed a significant difference in CoV_VD_ values between phases [*F*_(6, 67.19)_ = 3.92, *p* = 0.002]. *Post-hoc* tests showed that the CoV_VD_ value for the Stand_post_ was significantly higher compared to several positions during sedation and anaesthesia, namely, Sternal_sed_ [β = −3.77, SE = 0.95; *t*_(67.14)_ = −3.98, *p* = 0.003], Sternal_CMVhigh_ [β = −3.06, SE = 0.95; *t*_(67.14)_ = −3.23, *p* = 0.029], Sternal_spontETT_ [β = −3.00, SE = 0.96; *t*_(67.07)_ = −3.11, *p* = 0.041], and Sternal_spont_ [β = −3.80, SE = 0.96; *t*_(67.07)_ = −3.94, *p* = 0.003] (Table 1, Figure 3).

**Table 1:** Electric impedance tomography variables (mean and standard deviation) in alpacas during different phases of the anaesthetic procedure.

Variable	Stand_pre_	Sternal_sed_	Sternal_CMVhigh_	Sternal_CMVlow_	Sternal_spontETT_	Sternal_spont_	Stand_post_
CoV_RL_ (%)	50.7 ± 2.6	49.9 ± 2.1	48.3 ± 3.1	48.3 ± 2.5	48.3 ± 3.2	49.9 ± 3.2	48.7 ± 3.2
CoV_VD_ (%)	46.9 ± 3.2	45.5 ± 2.4^A^	46.2 ± 1.9^B^	47.5 ± 2.1	46.2 ± 1.7^C^	45.4 ± 2.8^D^	49.2 ± 3.3^ABCD^
ROI-R (%)	47.9 ± 7.5	50.1 ± 6.2	54.7 ± 9.5	54.7 ± 7.7	54.7 ± 10	49.6 ± 9.9	53.7 ± 9.5
ROI-L (%)	52.0 ± 7.5	49.8 ± 6.2	45.1 ± 9.5	45.2 ± 7.7	45.2 ± 10	50.3 ± 9.9	46.2 ± 9.5
TIV (AU)	1.0 ± 0.3	0.8 ± 0.2	0.8 ± 0.3	0.9 ± 0.2	0.8 ± 0.2	1.2 ± 0.5	1.2 ± 0.6
MIV (AU)	33.7 ± 16.7^ABCDEF^	14.8 ± 7.6^A^	10.6 ± 3.1^BGH^	11.7 ± 4.5^C^	13.2 ± 5.0^D^	17.5 ± 9.5^EG^	15.4 ± 5.3^FH^
EELI	27.2 ± 3.4	26 ± 3.1	28.0 ± 2.8	28.2 ± 2.9	26.7 ± 2.6	26.2 ± 2.8	26.6 ± 3.4
Tidal rate	34.2 ± 12^ABCDEF^	16.5 ± 4.9^A^	13 ± 3.8^B^	12 ± 3.3^C^	15.2 ± 2.7^D^	14 ± 2.7^E^	14 ± 4.8^F^
TI (s)	0.7 ± 0.2^ABCDEF^	1.5 ± 0.4^AG^	2.1 ± 0.5^B^	2.3 ± 0.9^CG^	2.0 ± 0.5^D^	2.2 ± 0.4^E^	1.8 ± 0.6^F^

CoV_RL_ and CoV_VD_, centre of ventilation right to left and ventral to dorsal, respectively; ROI-R and ROI-L, region of interest on the right and left, respectively; TIV, tidal impedance variation; AU, arbitrary units; EELI, End-Expiratory Lung Impedance; TI, Time in inspiration. Mean values sharing the same superscript letter (A, B, C, D, E, F, G, H) denote significant differences within each row (*P* < 0.05). Values sharing the same letter differ significantly, whereas values without a common letter do not differ significantly.

### 3.2 Region of interest right and left lung

The distribution of ventilation in the left as well as in the right lung remained consistent, with no statistically significant differences observed across any of the position changes [*F*_(6, 66.06)_ = 1.30, *p* = 0.269].

### 3.3 Tidal rate and TIV

The overall effect of phase on tidal rate was [*F*_(6, 72.00)_ = 20.43, *p* < 0.001], where *post-hoc* test showed that the tidal rate of Stand_pre_ showed a significantly higher value in comparison to all other positions {Sternal_sed_ [β = 17.75, SE = 2.40; *t*_(67.06)_ = 7.38, *p* < 0.001], Sternal_CMVhigh_ [β = 21.25, SE = 2.40; *t*_(67.06)_ = 8.84, *p* < 0.001], Sternal_CMVlow_ [β = 22.25, SE = 2.46; *t*_(67.45)_ = 9.04, *p* < 0.001], Sternal_spontETT_ [β = 18.98, SE = 2.46; *t*_(67.45)_ = 7.71, *p* < 0.001], Sternal_spont_ [β = 20.25, SE = 2.46; *t*_(67.45)_ = 8.19, *p* < 0.001], Stand_post_ [β = 20.25, SE = 2.53; *t*_(67.77)_ = 8.01, *p* < 0.001]} (Table 1).

An overall significant effect of position on TIV was observed [*F*_(6, 67.08)_ = 3.21, *p* = 0.007], while *post-hoc* pairwise comparisons were not statistically significant after correction for multiple testing.

### 3.4 MIV

Minute impedance variation (MIV) showed differences across the phases [*F*_(6, 67.05)_ = 23.58, *p* < 0.001], where in *post-hoc* analysis a higher Stand_pre_ was observed compared to all other phases {Sternal_sed_ [β = 0.84, SE = 0.11; *t*_(67.00)_ = 7.92, *p* < 0.001], Sternal_CMVhigh_ [β = 1.10, SE = 0.11; *t*_(67.00)_ = 10.37, *p* < 0.001], Sternal_CMVlow_ [β = 1.03, SE = 0.11; *t*_(67.03)_ = 9.53, *p* < 0.001], Sternal_spontETT_ [β = 0.93, SE = 0.11; *t*_(67.03)_ = 8.52, *p* < 0.001], Sternal_spont_ [β = 0.71, SE = 0.11; *t*_(67.03)_ = 6.57, *p* < 0.001], Stand_post_ [β = 0.75, SE = 0.11; *t*_(67.04)_ = 6.70, *p* < 0.001]}. A lower MIV was found in Sternal_CMVhigh_ compared to Sternal_spont_ [β = −0.38, SE = 0.11; *t*_(67.03)_ = −3.55, *p* = 0.012] and Stand_post_ [β = −0.35, SE = 0.11; *t*_(67.04)_ = −3.16, *p* = 0.036] (Table 1, Figure 3).

### 3.5 TI

**Figure 4:**
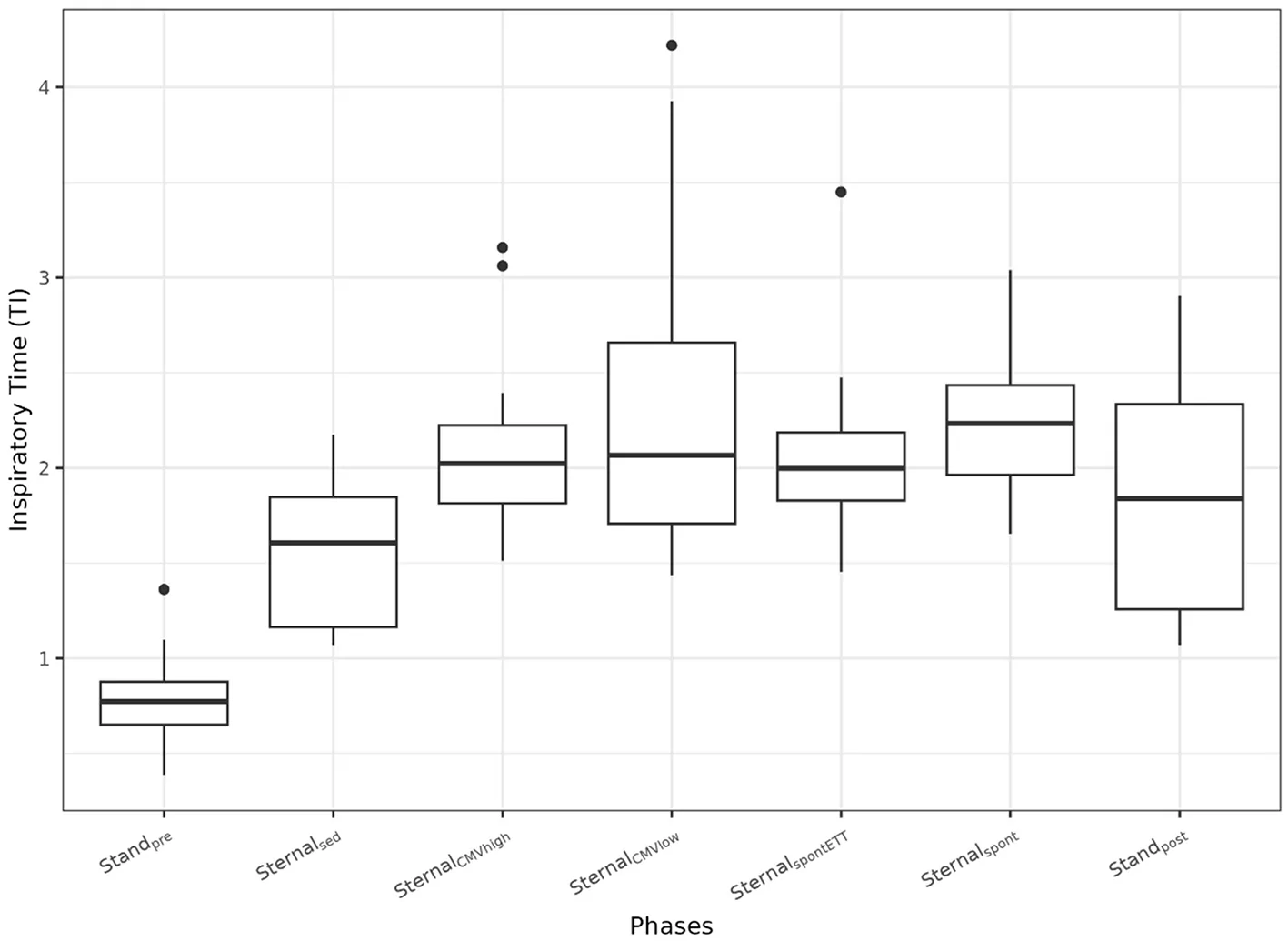
Boxplots represent the median and interquartile range of respiratory time displayed for six alpacas across all experimental phases, including sedation, controlled mechanical ventilation (head high/low), spontaneous breathing and post-anaesthesia, during two EIT measurement events. The x-axis represents the experimental phases: Stand_pre_, Standing pre-sedation; Sternal_sed_, Sternal recumbency after sedation; Sternal_CMVhigh_, Sternal recumbency controlled mechanical ventilation head high; Sternal_CMVlow_, Sternal recumbency controlled mechanical ventilation head low; Sternal_spontEIT_, Sternal recumbency spontaneous breathing with endotracheal tube; Sternal_spont_, Sternal recumbency spontaneous breathing without endotracheal tube; Stand_post_, Standing post anaesthesia. The y-axis shows the inspiratory time in seconds (s).

The phases had an overall significant impact on the Inspiration Time [*F*_(6, 67.30)_ = 11.37, *p* < 0.001], where in *post-hoc* analysis Stand_pre_ showed a shorter time compared to all other positions {Sternal_sed_ [β = −0.79, SE = 0.23; *t*_(67.03)_ = −3.45, *p* = 0.014], Sternal_CMVhigh_ [β = −1.36, SE = 0.23; *t*_(67.03)_ = −6.01, *p* < 0.001], Sternal_CMVlow_ [β = −1.58, SE = 0.23; *t*_(67.33)_ = −6.81, *p* < 0.001], Sternal_spontETT_ [β = −1.31, SE = 0.23; *t*_(67.33)_ = −5.65, *p* < 0.001], Sternal_spont_ [β = −1.48, SE = 0.23; *t*_(67.33)_ = −6.39, *p* < 0.001], Stand_post_ [β = −1.05, SE = 0.24; *t*_(67.52)_ = −4.42, *p* < 0.001]}. Furthermore, the animals showed a shorter TI in Sternal_sed_ compared to Sternal_CMVlow_ [β = −0.79, SE = 0.23; *t*_(67.33)_ = −3.40, *p* = 0.019] (Table 1, Figure 4).

### 3.6 EELI

**Figure 5:**
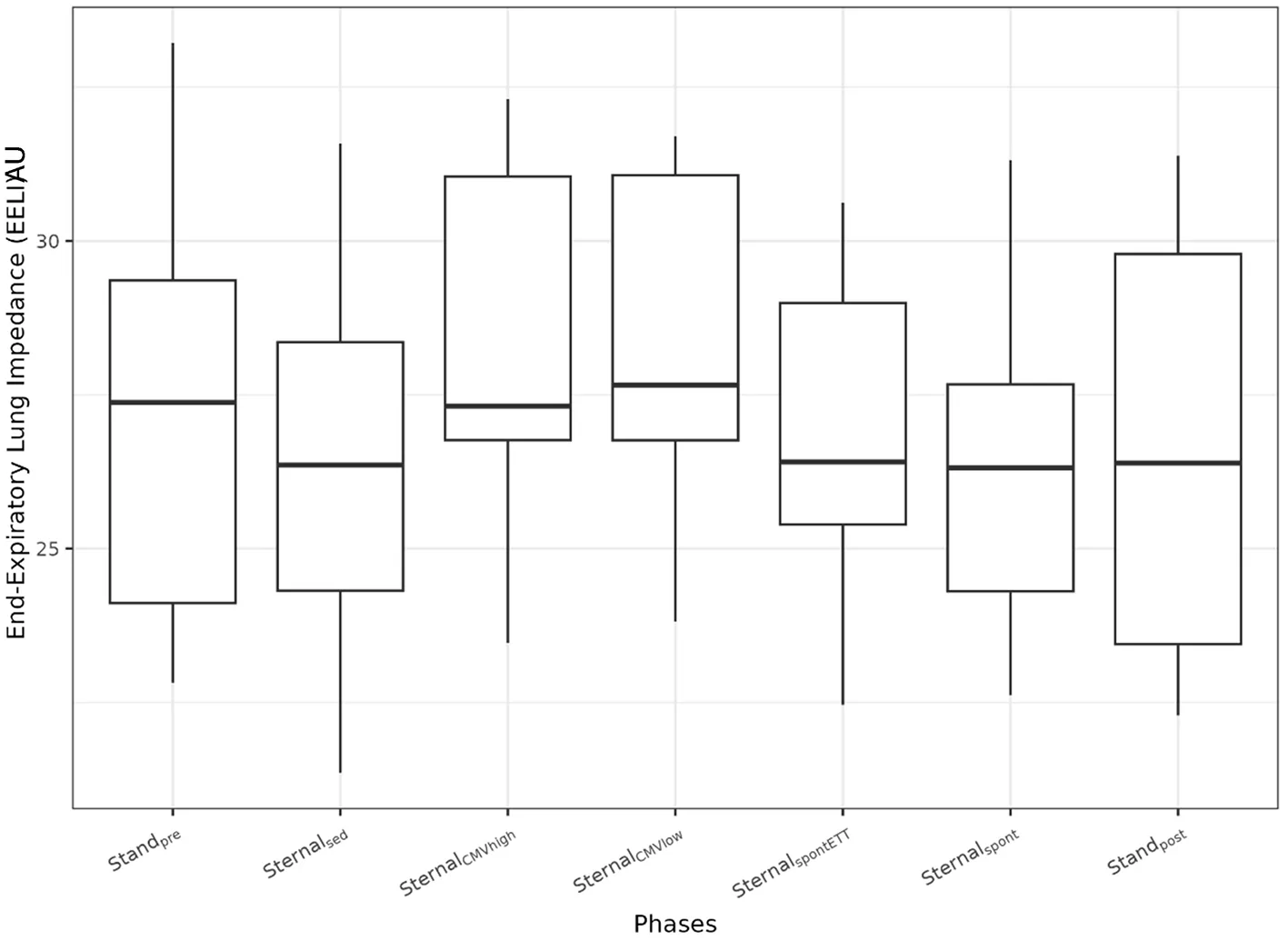
Boxplots represent the median and interquartile range for end-expiratory lung impedance displayed for six alpacas across all experimental phases, including sedation, controlled mechanical ventilation (head high/low), spontaneous breathing , and post-anaesthesia, during two EIT measurement events. The x-axis represents the experimental phases: Stand_pre_, Standing pre-sedation; Sternal_sed_, Sternal recumbency after sedation; Sternal_CMVhigh_, Sternal recumbency controlled mechanical ventilation head high; Sternal_CMVlow_, Sternal recumbency controlled mechanical ventilation head low; Sternal_spontEIT_, Sternal recumbency spontaneous breathing with endotracheal tube; Sternal_spont_, Sternal recumbency spontaneous breathing without endotracheal tube; Stand_post_, Standing post anaesthesia. The y-axis shows the end-expiratory lung impedance in arbitrary units (AU).

No statistically significant difference could be observed between the different positions for EELI [*F*_(6, 67.05)_ = 1.89, *p* = 0.094] (Table 1, Figure 5).

### 3.7 Ventilation asynchrony

Patient–ventilator asynchrony appeared more frequently during the Sternal_CMVhigh_ phase (6/12 observations) than during the Sternal_CMVlow_ phase (3/12 observations), based on qualitative assessment of ventilator waveforms, clinical observation, and EIT signal patterns.

## 4 Discussion

This study investigated the effects of sedation, general anaesthesia, and body/head positioning on ventilation distribution and respiratory parameters in alpacas using EIT.

Contrary to our hypothesis, body position and anaesthesia had minimal effects on ventilation distribution across all measured phases, which remained bilaterally balanced and predominantly localised to the dependent lung regions. Additionally, contrary to expectations, TIV and EELI remained stable across conditions, suggesting preserved tidal volume and functional residual capacity. As anticipated, a decrease in respiratory rate and MIV and TI was observed following sedation.

### 4.1 Effect of anaesthesia and body position on distribution of ventilation (CoV_*VD*_/CoV_*RL*_)

The distribution of ventilation in all alpacas remained in the dependent regions of the lungs throughout the entire procedure. In contrast to ruminants, where ventilation is centred in the non-dependent lung regions (10, 34), alpacas exhibited a distribution pattern more similar to that described in horses (35–37). This similarity may be partly explained by differences in lung morphology among these species. Ruminants possess a well-defined lung structure (11) whereas alpacas show minimal lobar separation, apart from the accessory lobe (38, 39). In the same way, horses display poor lobulation in their lung anatomy (11, 40), suggesting that the gross pulmonary structure of alpacas is anatomically closer to that of horses than to that of ruminants.

Following anaesthesia, during transition from sternal recumbency to Stand_post_, ventilation shifts dorsally toward the non-dependent regions of the lungs. This pattern likely reflects a rapid readjustment of abdominal organ pressure as the patient transitions from sternal recumbency.

A balanced right-to-left distribution of ventilation was observed throughout the study period, with no significant differences between body positions. These findings contrast with previous studies in calves, where approximately two-thirds of the inhaled air was directed to the right lung (19). A similar predominance of right lung ventilation has also been reported in horses and ponies (17, 37). This distribution pattern is largely explained by anatomical features; in both species, as in most mammals, the right lung is substantially larger than the left (41). Alpacas are no exception to this anatomical arrangement, although their unique gastrointestinal anatomy may play an important role in determining the distribution of ventilation. Interestingly, a study in pregnant ponies reported a balanced right-to-left ventilation distribution during gestation. The author of the study hypothesised that the gravid uterus exerts uniform pressure on the diaphragm, promoting a more central ventilation pattern (36). It is possible that the complex three-compartment gastrointestinal tract of alpacas exerts a similar effect, influencing the symmetrical distribution of ventilation observed in the present study.

### 4.2 Effect of anaesthesia and body position on TIV, Tidal Rate, MIV, TI, and EELI

Overall, sedation seems to have a major influence on various of the measured ventilation parameters. This observation was already described after xylazine sedation in horses, sheep, calves, and llamas (9, 11, 42–45).

#### 4.2.1 Tidal rate

A significant decrease in tidal rate was observed after premedication with xylazine ketamine and butorphanol compared with the unsedated condition. Consistent findings were reported by other authors after the use of xylazine (9, 10, 42–44). This decrease in tidal rate observed is consistent with the expected central depressant effects of α_2_-adrenergic agonists (46). However, the observed decrease in tidal rate is likely the result of the combined effects of xylazine, butorphanol, and ketamine, which may exert additive or synergistic influences on respiratory function.

Subsequently, following this initial decrease, no significant differences in tidal rate were detected among the other positions. Even after the recovery phase (Stand_post_), the tidal rate remained similar to that observed after sedation and lower than during Stand_pre_. The initially elevated tidal rate (Stand_pre_) may be attributed to the animals' exposure to a new environment (research area) and the EIT belt for the first time, which likely increased stress levels and, consequently, tidal rate. This finding is further supported by a lower tidal rate at Stand_pre_ during the second anaesthesia, with a median of 29 (18–36) breaths in alpacas already familiar with the environment, compared to a median of 40 (25–55) breaths observed during the first anaesthesia.

#### 4.2.2 TIV

In horses, xylazine administration has been reported to cause an initial reduction in tidal volume, followed by a subsequent increase over time (45). In contrast, sedation with xylazine in llamas has been associated with an overall increase in tidal volume (11).

In the present study, however, no differences in tidal impedance were observed among the various body positions. Moreover, no significant differences were detected between the sedation phase and the other experimental periods, contrasting with the results reported by previous studies.

#### 4.2.3 MIV

MIV closely followed the pattern observed for tidal rate, peaking in alpacas standing prior to sedation (Stand_pre_) compared with all other positions.

Although no significant difference was detected between the Sternal_CMVhigh_ and Sternal_CMVlow_ positions, MIV tended to be lower when the head was positioned higher, compared with values recorded during recovery (Sternal_spont_) and post-recovery (Stand_post_).

Interestingly, this observation coincided with a higher incidence of ventilator asynchrony in animals maintained in the elevated head position (6/12) compared with those in lower positions (3/12), despite adequate levels of anaesthesia. Several studies in human medicine have shown that patient–ventilator asynchrony can negatively impact ventilatory performance, leading to alterations in respiratory parameters (47–49).

This phenomenon may partially explain the reduced MIV observed during the Sternal_CMVhigh_ phase. However, this finding should be interpreted with caution, as additional factors, particularly sedation and anaesthesia, are known to markedly affect MIV.

#### 4.2.4 TI

As with MIV, TI was significantly shorter in Stand_pre_ than in all other positions, consistent with the higher tidal rate.

TI differed between Sternal_sed_ and Sternal_CMVlow_, with a longer TI observed in the latter, whereas no significant differences were detected between the two mechanically ventilated phases. Sternal_CMVlow_ appeared to improve synchrony between the animal and the ventilator, which may be associated with a more regular inspiratory pattern and, consequently, a longer TI. However, as asynchrony was assessed qualitatively and no direct relationship between asynchrony and TI was analysed, this interpretation should be approached with caution.

#### 4.2.5 EELI

EELI can be taken as a surrogate for FRC (14); a decrease in this parameter can, among others, indicate a reduction in lung compliance, increased abdominal pressure, pressure on the diaphragm, atelectasis, or a decrease in lung compliance (11).

No change in EELI was found between the different positions in the present study, an unexpected finding that suggests that functional residual capacity remained relatively stable across conditions.

Induction of general anaesthesia reduces FRC and promotes small airway collapse, mainly due to loss of respiratory muscle tone and visceral compression (50, 51). Application of PEEP increases FRC, helping prevent airway collapse and atelectasis (52). During the mechanical ventilation phases (Sternal_CMVhigh_, Sternal_CMVlow_), a PEEP of 5 cmH_2_O was applied, without any increase but also no decrease in EELI, and therefore in FRC. This finding indicates that this PEEP level was sufficient to keep lungs open in alpacas with healthy lungs.

### 4.3 Effect of head position during CMV

Alterations in head and neck position have been shown to immediately affect respiratory function in humans, primarily by reducing diaphragmatic efficacy. Reduced cervicothoracic mobility impairs normal respiratory mechanics by limiting diaphragmatic excursion and strength. Additionally, head rotation can interfere with breathing by altering diaphragmatic movement (53, 54).

Alpacas possess a particularly long neck, which is associated with an exceptionally long trachea. This anatomical feature could make these animals more susceptible to any alterations in ventilation due to head or neck position. Interestingly, in the present study, no significant differences were observed between the two head positions during mechanical ventilation. This lack of effect suggests that, as long as the neck and thereby the trachea, remain in a straight alignment, elevating or lowering the head and therefore changing the angle between head and neck has little influence on the distribution of alveolar ventilation. From a clinical perspective, these findings have practical relevance, particularly during dental and oral surgical procedures where variations in head position are common. However, the use of CMV may have influenced these findings and the results could vary during spontaneous ventilation.

### 4.4 CMV vs. spontaneous breathing with and without ETT

The absence of change in the EIT ventilation variables, including CoV, between CMV and spontaneous breathing was unexpected. Typically, during mechanical ventilation, the gas pushed into the lungs tends to move up, toward the non-dependent regions, as demonstrated in a study of anesthetised horses in dorsal recumbency (6). This redistribution has been attributed to the loss of dorsal diaphragmatic movement when switching from spontaneous ventilation to CMV (6, 12).

However, this expected shift was not observed in any of the alpacas included in this study. This is even more surprising as the ventilation in camelids relies predominantly on abdominal musculature, particularly the diaphragm and abdominal muscles, where the diaphragm is described as the primary muscle of ventilation in alpacas (55).

Alpacas have a diaphragm with a unique structure, including a small bone *(Os diaphragmaticum*) located in the *centrum tendineum* (55–57). This structure appears to be exclusive to camelids, including both Old World (*Camelus* spp.) (56) and New World (*Lama* and *Vicugna* spp.) species, and has not been reported as a normal anatomical feature in other mammals. Its exact function remains unclear, but it is thought to support the esophageal hiatus and foramen vena cava, maintaining their patency and protecting them from pressure exerted by the gastric viscera (55–59). Although one study in dromedaries described that *the Os diaphragmaticum* serves as an anchor for diaphragmatic muscle fibres, influencing how forces are transmitted across the diaphragm. By redistributing the pull from the lumbar muscular origin toward the anterior region, the bone spreads the mechanical load over a larger surface. Consequently, this portion of the diaphragm remains relatively fixed during breathing, in contrast to the more lateral regions, which must relax to accommodate respiratory movements (58). The predominant role of the diaphragm in ventilation, combined with these anatomical differences, may explain the relatively constant EIT variables observed across different positions in the alpacas in this study.

### 4.5 Measurement considerations of EIT, feasibility, and reliability

#### 4.5.1 Measurement considerations: rationale for time point selection

To obtain a representative set of artefact-free breaths for each phase, specific moments and durations within each time point were selected.

##### 4.5.1.1 Time-related considerations

A 5-min recording window was used in standing animals (Stand_pre_, Standp_ost_) to minimise measurement bias caused by movement artefacts or vocalisation, which can distort respiratory patterns. In contrast, during the sedated or anaesthetised phases, a 2-min recording period was sufficient to ensure the collection of adequate, artefact-free breaths.

##### 4.5.1.2 Position-related considerations

Sternal_sed_ values were recorded 10 min after achieving sternal recumbency, to allow lung adaptation and establish a stable ventilation distribution and ensure a reproducible sedation level. Measurements before CMV were not possible, as ventilation was initiated immediately after intubation to counteract potential atelectasis that occurs during possible apnoea phases following induction.

Positioning alpacas in a straight alignment under anaesthesia or sedation is challenging due to their long necks. It is also known that lowering the head after sedation can negatively affect several respiratory parameters (11, 45). To assess these effects, EIT data were collected in both head heights for 2 min after an equilibration phase of 28 min immediately before changing to the opposite head position.

To evaluate the influence of the endotracheal tube on breathing, EIT data were analysed for 2 min immediately before extubation and for another 2 min starting 2 min after extubation. To ensure a comparable depth of anaesthesia in both measurements, the closest possible measurement periods before and after extubation were chosen. The first 2 min following extubation had to be excluded because some animals exhibited coughing or slight head movements, which could introduce artefacts into the measurements.

#### 4.5.2 Feasibility

The present study demonstrates the practical feasibility of EIT in awake, sedated, and anaesthetised alpacas. Despite the dense fleece, the belt could be applied without difficulty, as the fleece was easily separated. The belt was well-tolerated; animals showed no signs of skin irritation and were able to stand comfortably while wearing the belt. Moreover, the use of a single belt size across all individuals suggests that the system is adaptable and straightforward to implement. Collectively, these results support the EIT belt as a safe and practical tool for monitoring in this setting, highlighting its potential for routine application.

#### 4.5.3 Repeatability

In the present study, a clear tendency toward repeatability of certain EIT-derived variables, such as TIV and MIV in Stand_pre_, was observed in individual animals between the first and second anaesthesia. However, due to the small sample size, no definitive statistical conclusions can be drawn.

Despite this limitation, the observation highlights an important area of potential clinical relevance. The repeatability of EIT measurements within the same patient over time is an aspect that warrants further investigation. Establishing reliable longitudinal consistency could support the use of EIT as a diagnostic tool, enabling clinicians to monitor the progression or resolution of respiratory diseases through serial EIT assessments in the same individual.

### 4.6 Limitations, clinical relevance & future

A limitation of this study is the relatively small sample size and the absence of a formal sample size calculation; however, each animal was measured twice, which enhances the robustness of the data. Another limitation is the lack of simultaneous spirometry, which would have allowed validation of EIT measurements but was avoided to minimise stress for the animals during the awake measurements. Additionally, changes in lung function measured with EIT have to be interpreted with caution as EIT captures only a cross-sectional lens-shaped area of the lung, meaning that derived parameters such as TIV and EELI do not represent absolute quantitative lung volumes however, they remain well suited for comparative analyses between positions and conditions.

From a clinical point of view, this study demonstrates that alpacas exhibit minimal recumbency and anaesthesia-related changes in the distribution of ventilation when maintained in sternal position with PEEP. Additionally, variations in head position, whether high or low, did not significantly affect ventilation. These findings suggest that, under these conditions, anaesthesia can be managed in alpacas without major impact on regional lung ventilation, supporting the use of sternal positioning and PEEP to maintain stable respiratory function during clinical procedures. Although endotracheal intubation was not required after sedation, the notable drop in minute ventilation observed in this study indicates that supplemental oxygen may be beneficial, particularly in compromised animals.

Future research should include a larger cohort to confirm and expand upon these findings and to correlate EIT-derived changes with gas exchange in each position. Furthermore, as this study only evaluated position changes between standing and sternal, future investigations should examine the effects of lateral and dorsal recumbency during anaesthesia to gain a more comprehensive understanding of positional impacts on ventilation in alpacas.

## 5 Conclusion

The changes in ventilation parameters observed in alpacas across different positions before, during, and after anaesthesia were lower than expected compared to other species. This relative stability may be largely due to the unique structure and function of their lung and diaphragm anatomy, which, along with the gastrointestinal tract, influences respiratory mechanics differently than in other mammals like ruminants and horses. Clinically, these findings suggest that changing body position from standing to sternal has less impact on ventilation distribution than anticipated. Sedation with xylazine ketamine and butorphanol altered tidal rate, minute impedance variation, and inspiratory time, but did not significantly affect tidal impedance variation or end-expiratory lung impedance. Overall, EIT proved to be a valuable tool for providing real-time insights into ventilation distribution and lung mechanics in standing, sedated, and anaesthetised alpacas.

## Data Availability

The original contributions presented in the study are included in the article/supplementary material, further inquiries can be directed to the corresponding author.
