# Correction: Effects of body positions and anaesthesia on the distribution of ventilation in alpacas (*Vicugna pacos*)

**DOI:** 10.3389/fvets.2026.1981704

**Published:** 2026-09-16

**Authors:** Frontiers Production Office

**Affiliations:** Frontiers Media SA, Lausanne, Switzerland

**Keywords:** alpaca, electrical impedance tomography, ventilation distribution, anaesthesia, body position

The funder University of Veterinary Medicine Vienna was erroneously omitted from the Funding statement. The correct **Funding** statement reads:

“The author(s) declared that financial support was received for this work and/or its publication. Open access funding provided by University of Veterinary Medicine Vienna.”

The original version of this article has been updated.

